# A prospective observational study of estimating drug related problems and clinical outcomes in subtypes of stroke patients

**DOI:** 10.1371/journal.pone.0295208

**Published:** 2024-01-02

**Authors:** Muhammad Ali, Muhammad Harris Shoaib, Shagufta Nesar, Muhammad Jamal, Sabiha Gul, Saira Shahnaz, Shoaib Nawaz, Quratulain Khan, Javaria Imran

**Affiliations:** 1 Department of Pharmaceutics, Faculty of Pharmacy and Pharmaceutical Sciences, University of Karachi, Karachi, Pakistan; 2 Department of Pharmaceutics, Faculty of Pharmacy, Salim Habib University, Karachi, Pakistan; 3 Jinnah College of Pharmacy Sohail University, Karachi, Pakistan; 4 University of Campania Luigi Vanvitelli, Naples, Italy; 5 Department of Pharmacy Practice, Nazeer Hussain University Karachi. Karachi, Pakistan; 6 Doctors Institute of Health Sciences, Sargodha, Pakistan; 7 College of Pharmacy, University of Sargodha, Sargodha, Pakistan; 8 Sindh Government Dispensary, Gharibabad District Central, Karachi, Pakistan; 9 NMC Specialty Hospital, Al Nahda, Dubai, UAE; Aga Khan University Hospital, PAKISTAN

## Abstract

**Background:**

Stroke is a neurological disease and a leading cause of mortality worldwide. Strokes mainly consist of two types: hemorrhage and ischemia. Stroke patients are being administered multiple drug therapy and are at risk of drug-related problems.

**Aim:**

To estimate drug-related problems (DRPs) and clinical end outcomes in hospitalized stroke patients.

**Methods:**

Current study was a multicenter, cross-sectional prospective observational study including 250 stroke patients admitted to tertiary care hospitals in Karachi, Pakistan. The study included all clinical subtypes of stroke patients i.e. Stroke, Ischemic stroke, Hemorrhagic stroke, CVA, and TIA. Associations among patient-clinical end outcomes and drug therapy-related variables like DRPs, mortality, and morbidity rates were estimated using Pearson’s chi-squared test. Statistical analysis was done by using SPSS software, version 25.

**Results:**

A total of 250 patients participated in this study suffering from different clinical subtypes of stroke i.e. Ischemic stroke, hemorrhagic stroke, TIA, and CVA, including 46% male and 54% female patients. The majority of patients’ stay at the hospital was between 1–10 days. The overall mortality rate in stroke patients was 51%. HAIs were observed in 70% of patients, HAIs faced by patients were SAP, CAP, UTI, sepsis, and VAP. Drugs were assessed according to NEML i.e. access group antibiotics, watch group antibiotics, reserve group antibiotics, statins, antiepileptics, and proton pump inhibitors. Majorly ceftriaxone was administered to 79% of patients, piperacillin-tazobactam to 52%, and cefixime to 48%, whereas meropenem was administered to 42% of patients along with vancomycin to 39% of total patients. A high mortality rate was observed in the case of *Klebsiella pneumoniae* and *Staphylococcus aureus* i.e. 78% and in the case of *streptococcus pneumoniae* 61% mortality rate was observed. Due to the presence of DRPs and various other clinical factors like comorbidities, DDIs, HAIs, administration of potentially nephrotoxic drugs, and administration of antibiotics without having CST, hospitalized stroke patients faced many problems.

**Conclusion:**

This study helped determine DRPs along with various clinical factors affecting the clinical end outcomes of patients suffering from any clinical subtype of stroke. Due to the enhancement in the evidence of the incidence of DRPs in tertiary care hospitals, pharmacist-led drug therapy review by interfering with doctors and other medical professionals at the patient bed site is needed and should be done to avoid any negative end outcomes and serious issues related to DRPs.

## Introduction

Stroke is a neurological disorder and a leading cause of mortality worldwide [1]. Strokes mainly consist of two types: hemorrhage and ischemia [2]. The clinical subtype of stroke that is the most common and leading cause of death in developing countries is ischemic stroke. [3]. Poor patient quality of life results from ischemic attack or minor stroke, which may also have long-term negative effects. [4]. Cerebrovascular accident (CVA) is also one of the leading socio-economic issues and causes high morbidity and mortality [5]. There are numerous stroke risk factors, such as hypertension, diabetes, migraines, excessive aging, etc. Women are more likely than males to experience a stroke. [6] Risk factors for stroke can be distributed into modifiable and non-modifiable risks. Modifiable risk factors include hypertension, smoking, diabetes mellitus, hyperlipidemia, heart failure, atrial fibrillation, alcohol consumption, positive family history, oral contraceptives, and polycythemia. Non-modifiable risk factors include age, gender (Males more than females), hereditary, and predisposition to vascular events such as myocardial infarction, stroke, or peripheral embolism. [7]. The prevalence of risk factors which will be estimated in the current study is very high, especially in Pakistan [8]. Stroke patients are being administered multiple drug therapy and are at risk of drug-related problems (DRPs), these DRPs are key determinants in this current study [9]. Along with drug therapy, stroke patients are also suggested for stroke rehabilitation in which stroke survivors relearn their lost skills [10]. DRPs can happen at any stage of the prescribing procedure and are rather common in hospitalized patients. The clinical pharmacist can make a significant contribution to the multidisciplinary team’s efforts to optimize pharmacotherapy for stroke patients [11].

Mainly stroke patients are administered many drugs like anti-platelet agents, statins, low molecular weight heparin, antihypertensives, oral hypoglycemic agents etc and for treatment of post-stroke infections various antibiotics are prescribed, multiple antibiotics were administered to stroke patients to treat infections, including ceftriaxone, levofloxacin, penicillin, and minocycline [12]. Post-stroke infections are very common in hospitalized patients and have negative outcomes on patients, commonly post-stroke infections are pneumonia and UTI [13]. Different scenarios for the occurrence of a post-stroke infection can be divided into three types: clinical factors, anatomical (stroke-related) factors, and immunological factors [14]. DRPs in stroke patients are high due to the administration of multiple drug therapy and in the presence of comorbidities and infections, DRPs are enhanced [15].

Identification and adjustment of the risk factors to reduce the prevalence of stroke is very critical, the frequency of stroke is very high in Pakistan and other developing countries [16]. A hospital-based study on stroke and its associated risk factors observed that in Pakistan, 31–40% of cases of stroke are due to cerebral hemorrhage and 60–69% are due to ischemia. In developed countries, about 85–90% of strokes are due to cerebral infarction, and 10–15% are due to intracranial hemorrhage. Hemorrhagic stroke is more prevalent in Asians [17]. The diagnostic procedures for stroke are the same in all adults, children, and geriatrics. CT (computerized tomography) of the brain is useful to differentiate hemorrhagic and non-hemorrhagic stroke in the very acute phase. MRI (magnetic resonance image) is also done in some cases. Intracranial as well as extracranial vessels are examined by an MRA (magnetic resonance arteriography) [18].

The primary goal of the current study was to estimate DRPs and other clinical factors that have an impact on stroke patients’ clinical end outcomes and they may prolong the length of hospital stay of stroke patients. Pharmaceutical Care Network Europe (PCNE) Classification Version 9.1, a classification system that was used for the estimation of DRPs in hospitalized stroke patients [19]. Multiple drugs belonging to different classes administered to stroke patients were also evaluated in terms of safety and efficacy. Frequency and prevalence of various types of microorganisms are involved in causing hospital acquired infections (HAIs) and post stroke infections among hospitalized stroke patients, as a result, this will be a significant factor influencing antibiotic therapy. For this reason, the antibiotic medication initiation and administration to hospitalised stroke patients was carefully examined.

## Methods

### Study design and sample

The current study was a multicenter, cross-sectional prospective observational study including 250 stroke patients admitted in one public and two private tertiary care hospitals. The study included all clinical subtypes of stroke patients i.e. Stroke, Ischemic stroke, Hemorrhagic stroke, Cerebrovascular accident (CVA) and Transient Ischemic attack (TIA) admitted either in the medical ward, neurology ward, intensive care units (Medical or Surgical ICUs) and isolation ward of public and private tertiary care hospitals of Karachi, Pakistan. The study sample included all those patients fulfilling inclusion and exclusion criteria. The research was conducted from October 2021 to December 2022. Patient/Informed consent form was taken from all the participants.

### Inclusion and exclusion criteria of patients

Patients admitted in the medical ward, neurology ward, intensive care Units (ICUs) both Medical or surgical and isolation ward suffering from any one clinical sub-type of stroke i.e. Ischemic stroke, Hemorrhagic stroke, Cerebrovascular accident (CVA) and Transient Ischemic attack (TIA). Special Personnel protective equipment (PPE) was used for the assessment of stroke patients data admitted in isolation wards [20, 21]. Patients from both sexes were included and classified into two categories as adults and geriatrics and patients having a total hospital stay length of fewer than 30 days were included in this study.

Patients less than 35 years of age, patients greater than 99 years of age, patients having a length of hospital stay more than 30 days and patients having a length of hospital stay of less than 2 days were excluded from this study.

### Ethical approval

Current research was approved by the Advanced Studies & Research Board (ASRB) of The University of Karachi, Karachi, Pakistan (Letter, ASRB/No./06314/Pharm.) and the Ethical Review Committee (ERC) of Sohail University, Karachi, Pakistan (Letter, Protocol I #:000160/22). After getting the approval, written informed consent was taken from all the participants and then patient data was collected from affiliated teaching hospitals of the University of Karachi, Karachi, Pakistan, and Sohail University, Karachi, Pakistan.

### Patient data collection procedure and clinical assessment

Data of patients were collected from patient’s daily data profiles using an adapted checklist. The age of patients, sex, clinical end outcomes, Clinical subtype of stroke, hospital stay, and hospital sector were recorded. Other data variables include hospital-acquired infections (HAIs), culture sensitivity tests (CST), drug therapy (antibiotics, statins, anticoagulants, antiplatelets, antivirals, antipyretics, antifungals, antiepileptics, antihypertensives, oral hypoglycemics, PPIs, NSAIDs etc), lab diagnostics and DRPs were also noted using adapted check list [22]. Kidney function was assessed using the Cockroft-Gault equation [23]. Potentially nephrotoxic drugs administered among stroke patients whose kidneys are compromised were also assessed. Drug-drug interactions (DDIs) were checked using two software i.e. Stockleys’s drug interaction [24, 25] and Medscape drug interaction checker [26]. Clinical end outcomes were set as expired or discharge of patient on the last day of patient stay at tertiary care hospital. Empirical or rational use of antibiotic therapy for post-stroke infections and HAIs was evaluated using the results of CST performed on patients [27] and by using the American Society of Infectious Diseases (IDSA) guidelines [28, 29]. Antibiotics and other drug therapy administered in hospitalized stroke patients were also estimated by utilizing the National essential medicine list (NEML) of Pakistan [30].

### Study tool (evidence and Indication based clinical assessment checklist)

After doing an extensive medical literature review related to all clinical subtypes of stroke and having adapted some points from PCNE classification, an evidence, and indication-based clinical assessment checklist was developed. Face validation was also done by consulting with specialized medical professionals and experts including infectious disease specialists, clinical pharmacists and physicians [31–33],It was then employed as a study tool for this current research. This tool was used to estimate patients and to determine DRPs, their causes (possible causes or reasons for potential problems) [19, 22]. Patients were individually evaluated using the developed checklist along with clinical end outcomes in terms of the safety and efficacy of the drug therapy administered to hospitalized stroke patients. Variables of the patients which were studied included age, gender, clinical end outcome, type of stroke, hospital stay, and sector of the hospital. Other data information included HAIs like Stroke associated pneumonia (SAP), fever, community-acquired pneumonia (CAP), ventilator-associated pneumonia (VAP), sepsis, urinary tract infections (UTI) etc, CST, drug therapy administered (antibiotics, statins, anticoagulants, antiplatelets, antivirals, antipyretics, antifungals, antiepileptics, antihypertensives, oral hypoglycemics, PPIs, NSAIDs etc), lab diagnostics like Complete blood count (CBC), CST, Serum creatinine, Lipid profile and electrolytes etc. Status of Computed tomographic (CT) scan, Cerebral spinal fluid (CSF), and Magnetic resonance imaging (MRI) advised by medical practitioner were also seen.

### Statistical analysis

Stroke’s patient data collected through the study tool was duplicated/transferred to SPSS (Statistical Package for the Social Sciences), Version 25. The statistical procedure was performed, first a descriptive analysis in which patient clinical scenarios and various variables were estimated for their respective frequencies, percentages, and measure of central tendencies. Secondly, inferential statistics were employed to establish the associations among patient-clinical end outcomes and drug therapy-related variables like DRPs and mortality and morbidity rates using Pearson’s chi-squared test. confidence interval of 95%, P-value <0.05 was considered statistically significant.

## Results

**Fig 1:**
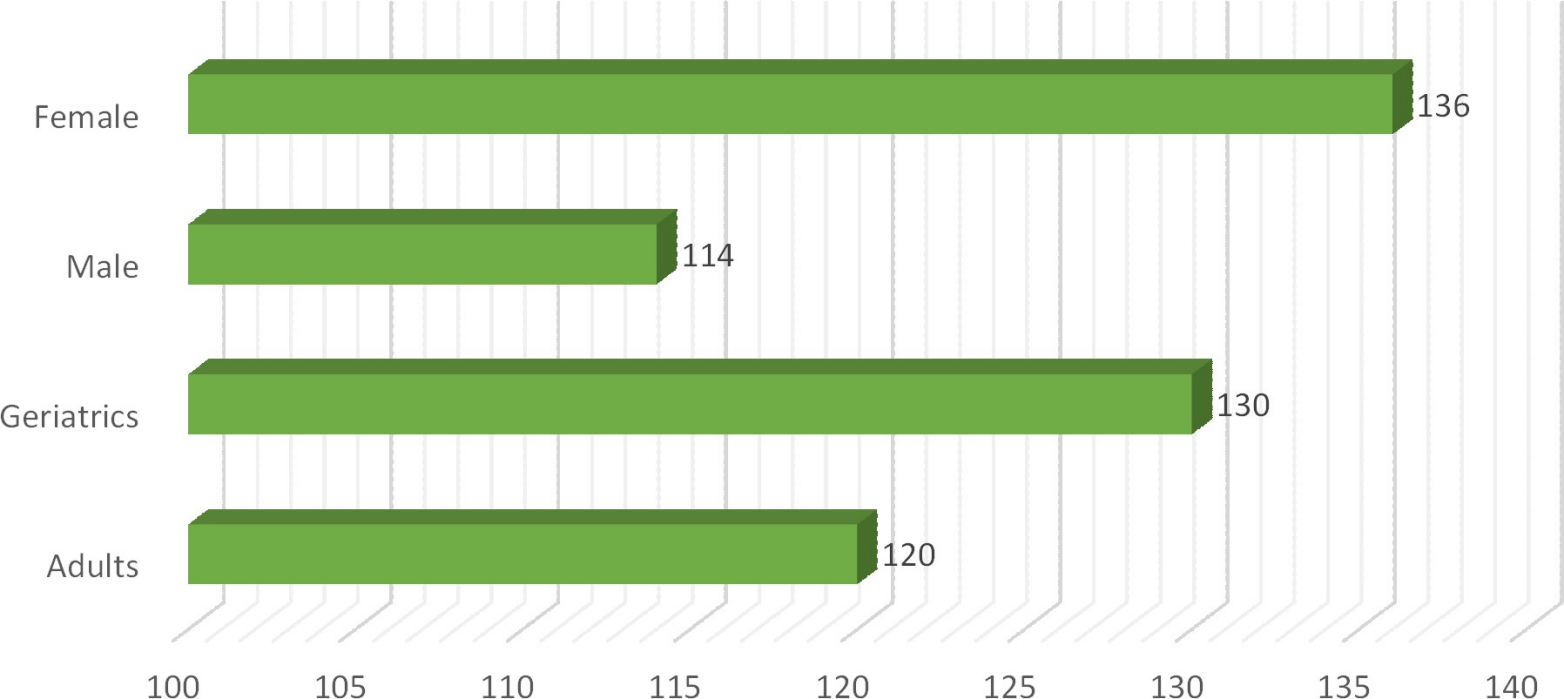
Frequency of Age groups and sex of hospitalized stroke patients.

**Fig 2:**
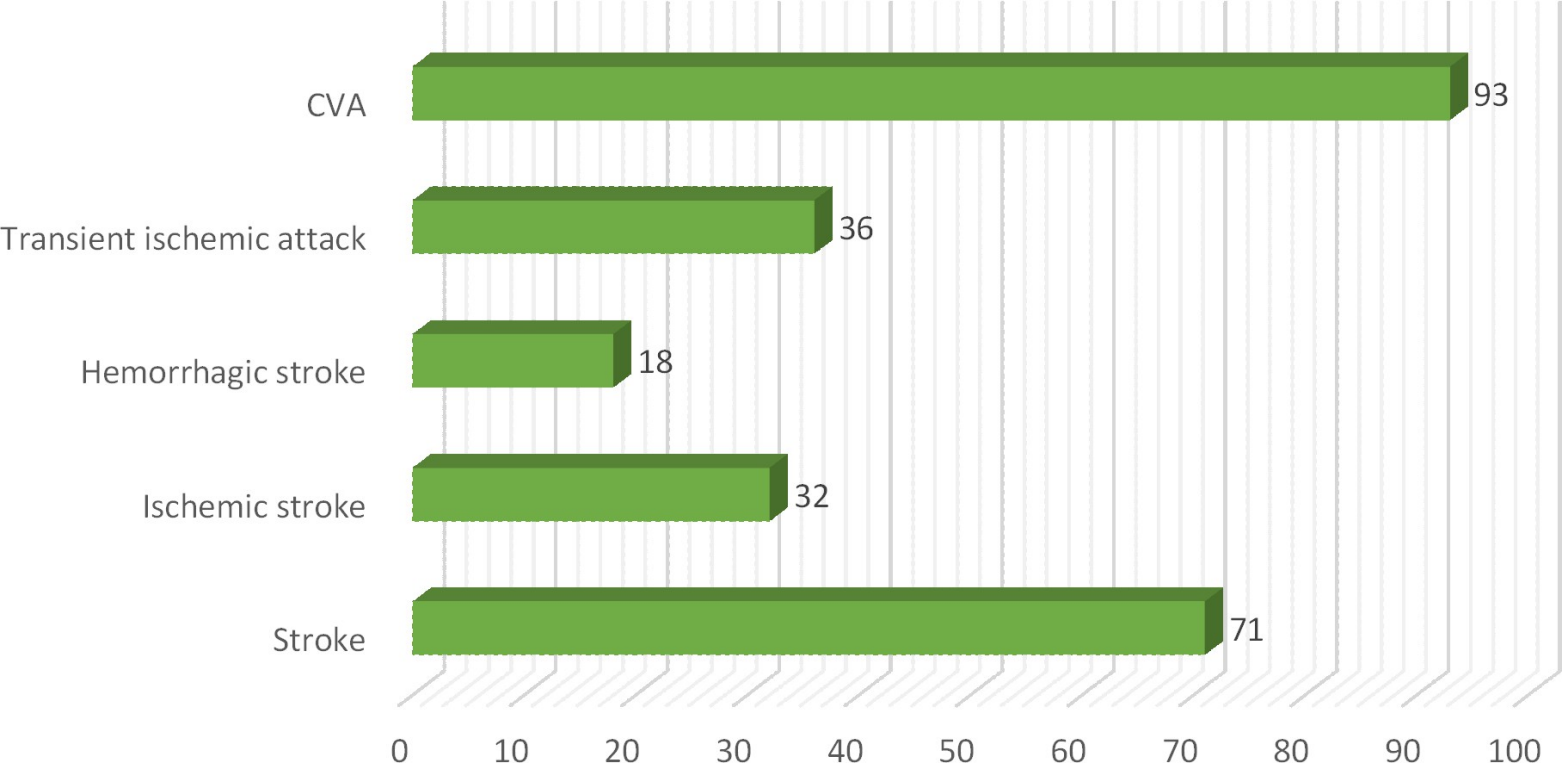
Frequency of different clinical subtypes of stroke according to primary diagnosis.

**Fig 3:**
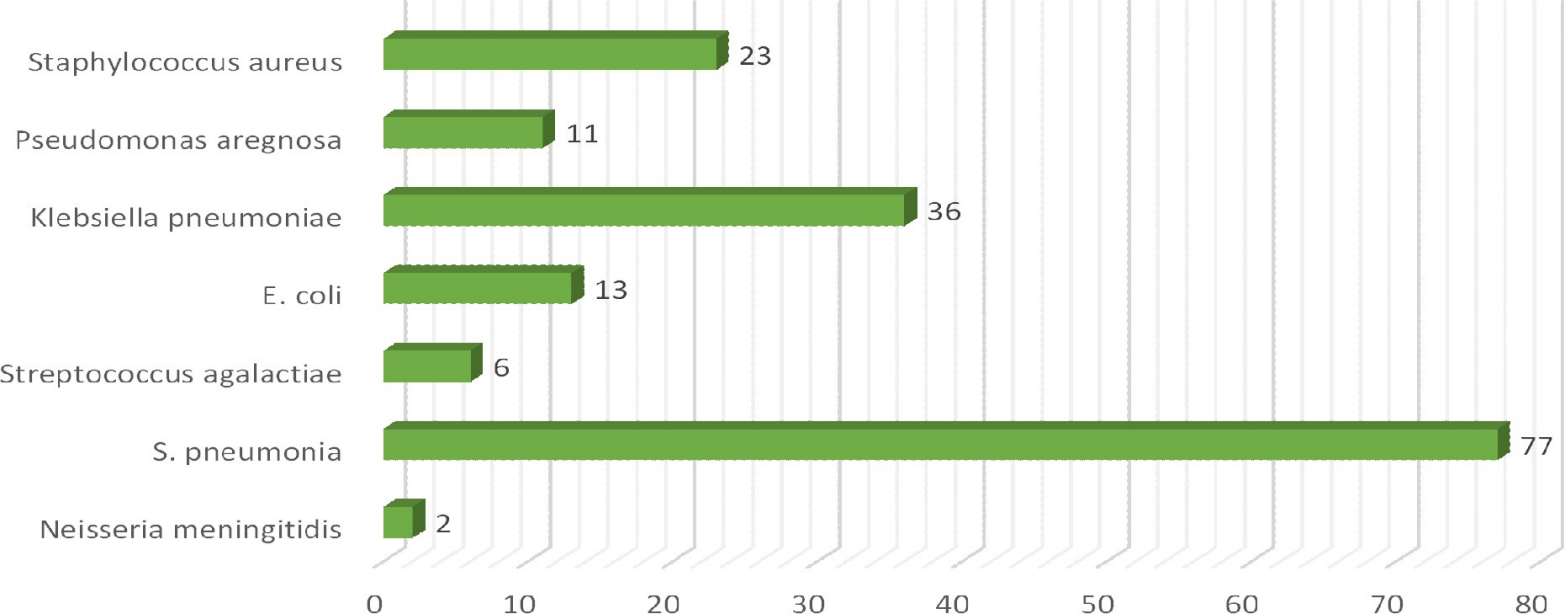
Frequency of different microorganisms causing hospital acquired infections and post stroke infections among hospitalized stroke patients.

**Fig 4:**
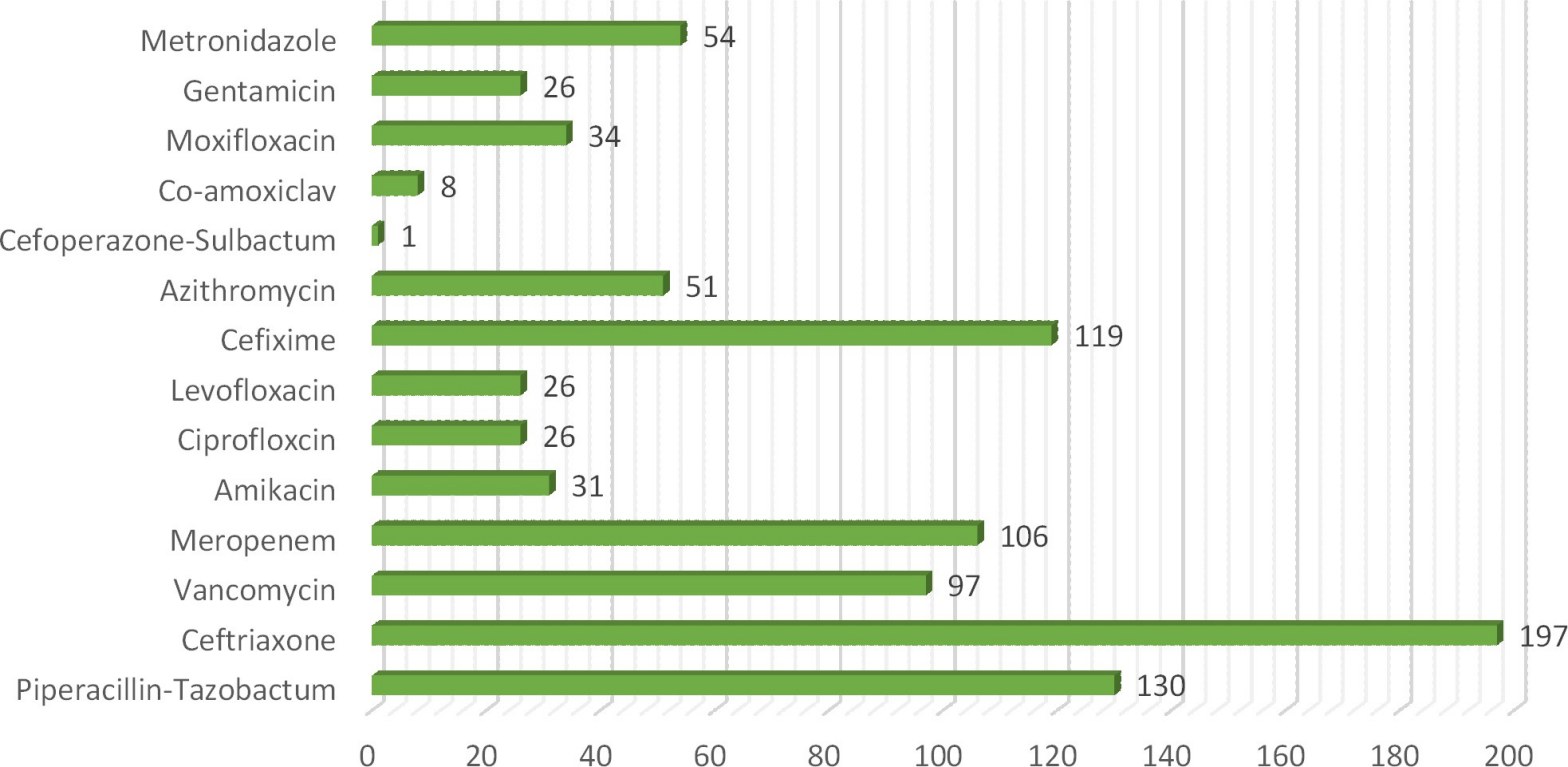
Frequency of different antibiotics administered for treatment of hospital acquired infections and post stroke infections among hospitalized stroke patients.

**Fig 5:**
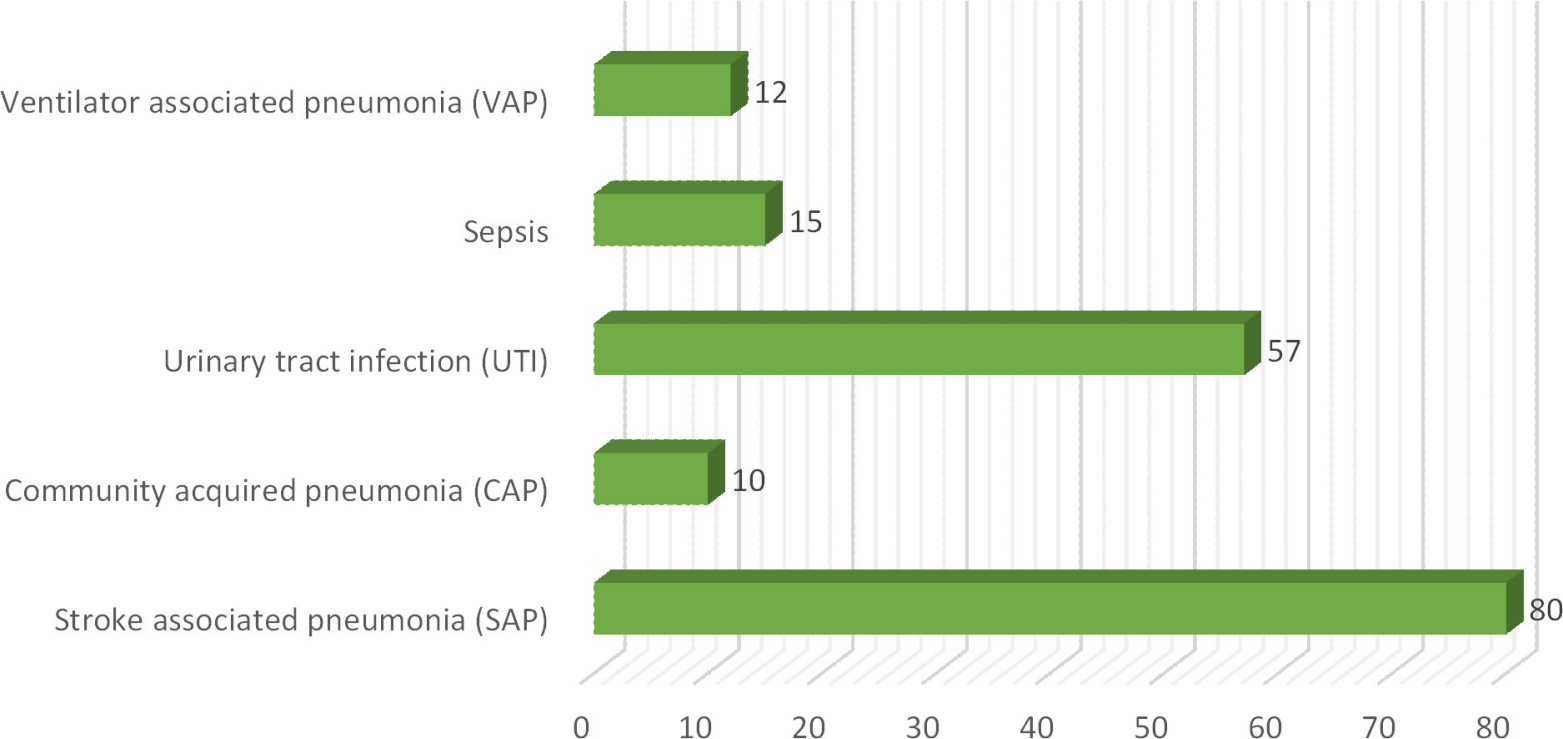
Frequency of different hospital acquired infections and post stroke infections among hospitalized stroke patients.

**Fig 6:**
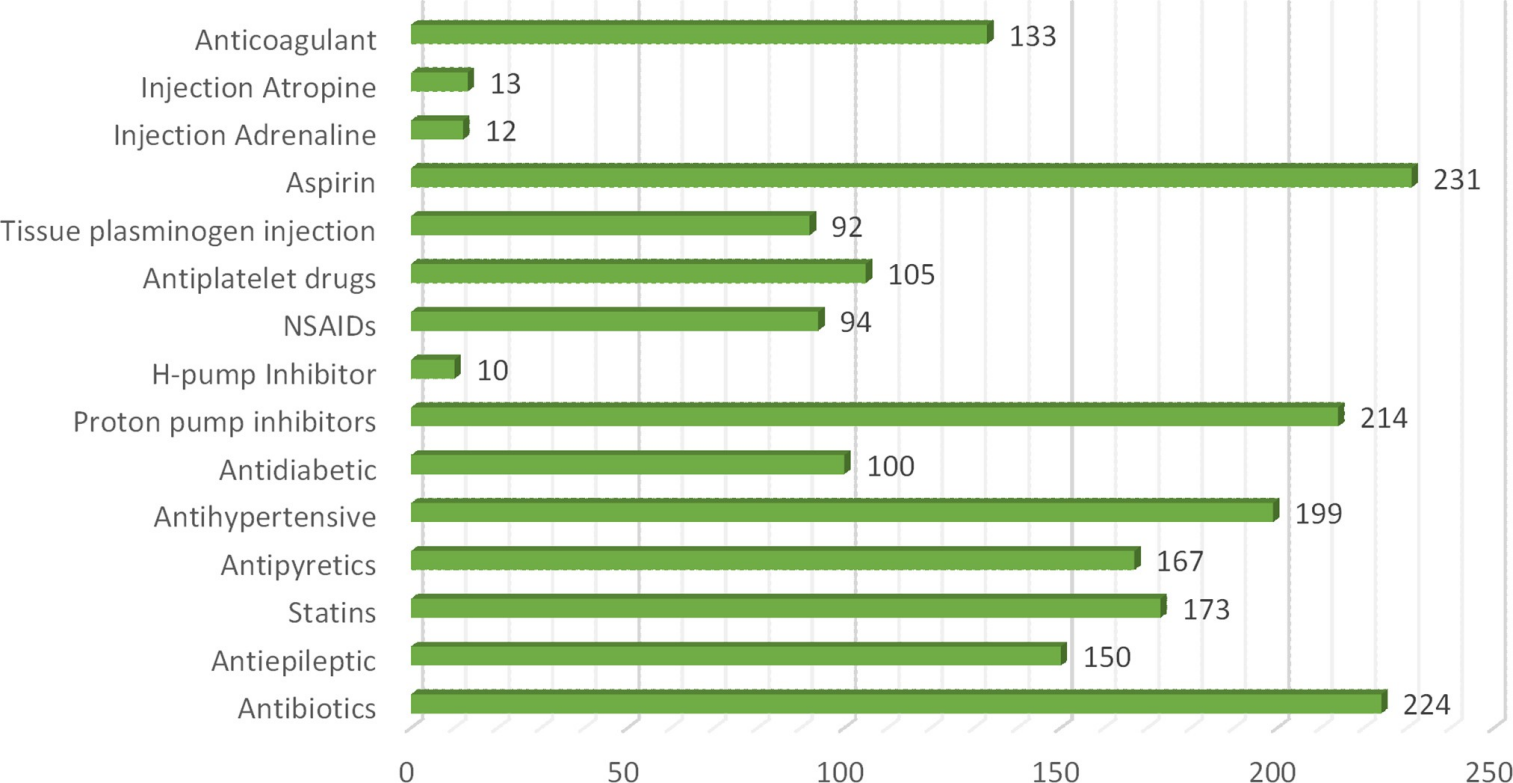
Frequency of different classes of drugs administered to hospitalized stroke patients.

In this current study total of 250 patients participated suffering from different clinical subtypes of stroke i.e. Ischemic stroke, TIA, hemorrhagic stroke, and CVA, including 46% male and 54% female patients. Among both male and female stroke patients, 120 patients were adults and 130 patients were geriatrics as mentioned in (Fig 1). The majority of patients stay at the hospital between 1–10 days. The frequency of different types of stroke according to primary diagnosis is illustrated in (Fig 2). Different microorganisms causing hospital-acquired infections (HAIs) and post-stroke infections among hospitalized stroke patients are demonstrated in (Fig 3). Antibiotics were prescribed and administered to patients having HAIs and post-stroke infections, as mentioned in (Fig 4). HAIs faced by patients were stroke-associated pneumonia (SAP), community-acquired pneumonia (CAP), urinary tract infection (UTI), sepsis, and ventilator-associated pneumonia (VAP), shown in (Fig 5). Due to the presence of HAIs, post-stroke infections, and comorbidities, multiple drug therapy was administered to hospitalized stroke patients, in (Fig 6), frequency of different classes of drugs administered to stroke patients is demonstrated.

Different classes of drugs administered to hospitalized stroke patients according to NEML and their statistical association with clinical end outcomes of hospitalized stroke patients is done, see (Table 1). The significant statistical association between drug-drug Interactions (DDIs) and clinical end outcomes of hospitalized stroke patients, see (Table 2). The association between DDIs, HAIs, and predisposing factors/comorbidities among hospitalized stroke patients is explained, see (Table 3). The statistical association between various clinical situations faced by hospitalized stroke patients and their clinical end outcomes, see (Table 4). As in this study estimation of DRPs was done, and the estimation of potentially nephrotoxic drugs given to renally compromised hospitalized stroke patients and their statistical association is done, see (Table 5). Tables 6 & 7 were adapted from Pharmaceutical Care Network Europe (PCNE) Classification Version 9.1, which demonstrate the statistical Association between DRPs and clinical end outcomes among hospitalized stroke patients and the statistical association between DRPs and hospital-acquired infections among hospitalized stroke patients respectively.

**Table 1:** Different classes of drugs administered to hospitalized stroke patients according to National essential medicine list (NEML) of Pakistan and their statistical association with clinical end outcomes of hospitalized stroke patients.

Drugs administered to hospitalized stroke patients according to NEML	Clinical End Outcomes		*p*-value
	Expired	Discharged	
**Statins**	87	60	**0.003**
**Antiepileptics**	86	63	**0.012**
**Proton pump inhibitors**	117	101	**0.041**
**Access group Antibiotics**	70	71	0.576
**Watch group Antibiotics**	123	86	**0.000**
**Reserve group Antibiotics**	92	49	**0.000**

**Table 2:** Statistical association between Drug-drug Interactions (DDIs) and Clinical end outcomes of hospitaized stroke patients.

**Drug-Drug Interactions (DDIs) faced by hospitalized stroke patients**	**Clinical end outcomes of hospitalized stroke Patients**		**Total (n)**	***p*-value**
	**Expired**	**Discharged**		**0.045**
**Major DDIs**	92	73	165	
**Minor DDIs**	36	49	85	

**Table 3:** Statistical association between DDIs, HAIs, Predisposing factors/comorbidities among hospitalized stroke patients.

Various Clinical Variables	DDIs	Frequency (n)	*p*-value
**Hospital Acquired Infections (HAIs)**	Major DDIs	129	**0.000**
	Minor DDIs	45	
**Predisposing factors and Comorbidities (Mainly Hypertension, Diabetes & extreme age)**	Major DDIs	151	**0.002**

**Table 4:** Statistical association between various clinical situations faced by hospitalized stroke patients and their clinical end outcomes.

Various Clinical Variables/Situations	Clinical end outcomes of hospitalized stroke patients		Total (n)	*p*-value
	Expired	Discharged		
**HAIs**	118	56	174	**0.000**
**Major DDIs**	92	73	165	**0.045**
**Comorbidities**	114	87	201	**0.001**
**No CST**	97	107	204	**0.015**
**Covid-19 Virus**	25	19	44	0.411

**Table 5:** Estimation of potentially nephrotoxic drugs given to renally compromised hospitalized stroke patients.

Different drugs administered to hospitalized stroke patients	Estimation and frequency of Renally compromised hospitalized stroke patients (n)		*p*-value
	Yes	No	
**Piperacillin-Tazobactum**	50	80	**0.047**
**Ceftriaxone**	71	126	**0.035**
**Vancomycin**	39	58	**0.047**
**Meropenem**	43	63	**0.025**
**Amikacin**	10	21	0.945
**Ciprofloxcin**	12	14	0.125
**Levofloxacin**	10	16	0.516
**Cefixime**	37	82	0.584
**Azithromycin**	13	38	0.128
**Cefoperazone-Sulbactum**	01	00	0.183
**Co-amoxiclav**	04	04	0.403
**Moxifloxacin**	17	17	**0.057**
**Gentamicin**	10	16	0.516
**Omeprazole**	73	141	0.281
**Levetiracetum**	38	59	0.087
**Acyclovir**	11	27	0.583
**Dexamethasone**	27	41	0.155
**Diclofenac sodium**	10	35	0.095
**Paracetamol**	64	103	**0.008**
**Ibuprofen**	16	39	0.507

**Table 6:** Statistical Association between DRPs and clinical end outcomes among hospitalized stroke patients.

Primary Domain	Causes	Frequency of DRPs (n)	Clinical end outcomes		*p*-value
			Expired	Discharge	
**Drug Selection**	Inappropriate drug according to guidelines/formulary	86	62	24	**0.000**
	Inappropriate combination of drugs, or drugs and herbal medications, or drugs and dietary supplements	102	56	46	
	Appropriate drug according to guidelines/formulary	62	10	52	
**Dose Selection**	Dosage regimen not frequent enough	132	70	62	**0.000**
	Dosage regimen too frequent	53	18	35	
	Dose timing instructions wrong, unclear or missing	12	10	02	
**Treatment Duration**	Duration of treatment too short	90	44	46	**0.000**
	Duration of treatment too long	87	59	28	
	Proper Duration of treatment	73	25	48	
**Dispensing**	Prescribed drug not available	47	22	25	**0.002**
	Necessary information not provided or incorrect advice provided	120	55	65	
**Drug use process**	Inappropriate timing of administration or dosing intervals	110	61	49	**0.000**
	Wrong drug administered	70	48	22	
	Appropriate timing of administration or dosing intervals	65	17	48	

**Table adapted from Pharmaceutical Care Network Europe (PCNE) Classification Version 9.1** ^**45**^

**Table 7:** Statistical Association between DRPs and hospital-acquired infections among hospitalized stroke patients.

Primary Domain	Causes	Frequency of DRPs (n)	Hospital acquired Infections		*p*-value
			Yes	No	
**Drug Selection**	Inappropriate drug according to guidelines/formulary	86	80	06	
	Inappropriate combination of drugs, or drugs and herbal medications, or drugs and dietary supplements	102	74	28	**0.000**
	Appropriate drug according to guidelines/formulary	62	20	42	
**Dose Selection**	Dosage regimen not frequent enough	132	93	39	**0.000**
	Dosage regimen too frequent	53	31	22	
	Dose timing instructions wrong, unclear or missing	12	06	06	
**Treatment Duration**	Duration of treatment too short	90	63	27	
	Duration of treatment too long	87	67	20	0.072
	Proper Duration of treatment	73	44	29	
**Dispensing**	Prescribed drug not available	47	31	16	**0.003**
	Necessary information not provided or incorrect advice provided	120	80	40	
**Drug use process**	Inappropriate timing of administration or dosing intervals	110	84	26	**0.000**
	Wrong drug administered	70	60	10	
	Appropriate timing of administration or dosing intervals	65	26	39	

**Table adapted from Pharmaceutical Care Network Europe (PCNE) Classification Version 9.1** ^**45**^

## Discussions

In this current study, DRPs and clinical end outcomes in patients suffering from any clinical subtype of stroke were estimated. Different classes of drugs administered to hospitalized stroke patients according to NEML of Pakistan and their statistical association with clinical end outcomes of hospitalized stroke patients were discussed in Table 1. Drugs which were assessed according to NEML were access group antibiotics, watch group antibiotics, reserve group antibiotics, statins, antiepileptic drugs and proton pump inhibitors [35, 36]. CST was initiated in only 18% hospitalized stroke patients, use and administration of antibiotics is based on CST, so in our study it was observed that due to unavailability or not having CST, a high mortality rate of 96% was observed in those stroke patients who were administered watch group antibiotics and 72% mortality rate was observed in those stroke patients having administration of reserve group antibiotics and due to presence of major DDIs as multiple drug therapy was administered to stroke patients, this rate is much higher than previous studies conducted in developing countries [37]. No procedure or rule was followed for de-escalation or avoiding unnecessary antibiotics was done as mentioned in one of the previous research study on rational use of antibiotics [29].

In current study, HAIs were found in 70% patients, among these patients having HAIs major DDIs were observed in 74% patients [37]. A high mortality rate of 92% was observed in those stroke patients who were facing HAIs as compared to previous studies this is very high mortality rate [38, 39]. Comorbidities were found in 87% of patients having HAIs, hence patients were prone to more compromised state due to presence of various above factors [15]. DDIs are evident due to multiple drug therapy, 84% major DDIs were found in those patients having comorbidities specially hypertension and diabetes [40, 41]. In this study 44 patients were diagnosed with Covid-19 virus while their stay at the hospital and hence shifted to isolation ward, some previous studies also suggested that patients having neurological diseases are already compromised so they are prone to covid-19 virus and hence poor prognosis [42, 43].

Many antibiotics were prescribed and administered to patients having HAIs and post stroke infections. Majorly ceftriaxone was administered to 79% patients, pipercillin tazobactum 52%, cefixime 48%, whereas meropenem was administered in 42% patients along with vancomycin in 39% of total patients. Meanwhile other antibiotics administered included ciprofloxacin and levofloxacin 10%, metronidazole 22%, gentamicin 10%, cefoperazone-sulbactum 04%, amikacin 12%, moxifloxacin 14%, linezolid 2%, co-amoxiclav 3.2% and azithromycin 20%, previous study also demonstrated the use of antibiotics for treatment of post stroke infections [14] In current study, it was observed that 33% of the patients were renally compromised, and hence they require careful administration of potentially nephrotoxic drugs because due to presence of comorbidities, DDIs, extreme age and HAIs chances of drug induced nephrotoxicity are enhanced, Table 5 showed the frequency and estimation of potentially nephrotoxic drugs given to renally compromised patients. Values of serum creatinine were used to estimate or assess the renal compromise stroke patients, cockroft-Gault equation was used in this regard [23]. Therapeutic drug monitoring or dose adjustment is required for renally compromised patients for safe and effective treatment. [44]. Many microorganisms were involved in causing HAIs and post stroke infections. High mortality rate was observed in case of *klebsiella pneumoniae* and *staphylococcus aureus* 78% and in case of *streptococcus pneumoniae* 61% expiries were observed. Various HAIs were observed in stroke patients, majority of patients were facing SAP 46%, UTI was present in 33% patients [45, 46]

DRPs were estimated in hospitalized stroke patients by adapting some points from PCNE classification V. 9.1 and their statistical associations were evaluated against clinical end outcomes and HAIs of patients, all the results and associations were statistically significant as mentioned in Tables 6 & 7 [47]. This Present study is the first study in best of our knowledge to use PCNE classification V 9.1. for estimation of DRPs in hospitalized stroke patients, previous studies used old versions or volumes [9, 48]. It was observed that due to presence of DRPs and various other clinical factors like comorbidities, DDIs, HAIs, administration of potentially nephrotoxic drugs and administration of antibiotics without having CST, hospitalized stroke patients faced many problems, they not only have a bad impact on their quality of life but they also have a huge impact on their morbidity and mortality rate. The main concern is that DRPs are common in stroke patients, therefore clinical pharmacists and other healthcare team members should work collectively to reduce and prevent DRPs with the focus on performing medication review, dosage adjustment, and proper drug selection according to guidelines for stroke patients [49].

## Conclusion

The present study helped determine DRPs along with various clinical factors affecting the clinical end outcomes of patients suffering from stroke. After estimation of DRPs and other related clinical factors, it was concluded that this study will help health care teams to follow up or treat stroke patients according to guidelines. Pharmacist intervention will pinpoint and resolve DRPs to amend drug therapy. Due to the enhancement in the evidence of the incidence of DRPs in tertiary care hospitals, pharmacist-led drug therapy review by interfering with doctors and other medical professionals at the patient bed site is needed and should be done to avoid any negative end outcomes and serious issues related to DRPs along with irrational use of antibiotics as they have an impact on patient quality of life, extended hospital stay, expensive treatment, and mortality rate in the developing countries. Whereas focused education should be given to healthcare teams regarding the importance of CST before initiation and administration of antibiotic therapy in post-stroke infection patients.

## Supporting information

S1 Data (ZIP)

## Abbreviations

^1^HAIs: Hospital acquired infections
^2^DRPs: Drug related problems
^3^CST: Culture sensitivity test
^4^CAP: Community acquired pneumonia
^5^DDIs: Drug-drug interactions
^6^NEML: National essential medicine list of Pakistan
^7^DRAP: Drug regulatory of Pakistan
^8^SAP: Stroke associated pneumonia
^9^VAP: ventilator associated pneumonia
